# In Situ Synthesis of Silicon–Carbon Composites and Application as Lithium-Ion Battery Anode Materials

**DOI:** 10.3390/ma12182871

**Published:** 2019-09-05

**Authors:** Dae-Yeong Kim, Han-Vin Kim, Jun Kang

**Affiliations:** 1 Division of Marine Engineering, Korea Maritime and Ocean University, 727 Taejong-ro, Yeongdo-gu, Busan 49112, Korea; 2 Department of Electrical and Electronic Engineering, Tokyo Institute of Technology, 2-12-1 Ookayama, Meguro-ku, Tokyo 152-8550, Japan

**Keywords:** silicon–carbon composites, lithium-ion battery, anode materials, bottom-up approach, plasma in solution

## Abstract

Silicon can be used in a variety of applications. Particularly, silicon particles are attracting increased attention as energy storage materials for lithium-ion batteries. However, silicon has a limited cycling performance owing to its peeling from the current collector and the volume expansion that occurs during alloying with lithium in the charging process. Significant contributors to this problem are the even distribution of silicon nanoparticles within the carbon matrix and their deep placement in the internal structure. In this study, we synthesized silicon nanoparticles and carbon materials via a bottom-up approach using a new method called plasma in solution. Silicon nanoparticles and the carbon matrix were synthesized in a structure similar to carbon black. It was confirmed that the silicon particles were evenly distributed in the carbon matrix. In addition, the evaluation of the electrochemical performance of the silicon–carbon matrix (Si–C) composite material showed that it exhibited stable cycling performance with high reversible capacity.

## 1. Introduction

Silicon nanoparticles (NPs) have been considered unwanted contaminants that are formed as the by-product of semiconductor fabrication [1] during the thermal cracking of silane (SiH_4_). Nowadays, silicon can be used in a variety of potential applications as a new material when it is synthesized to have a certain size and shape [2,3,4].

Silicon nanoparticles have been extensively used in lithium-ion battery anodes [5], hydrogen generation [6], photodetectors [7], photocatalysts [8], and sensing [9]. Specifically, silicon particles have been attracting increased attention as energy storage materials for lithium-ion batteries. Generally, in commercial lithium-ion batteries, graphite is the most commonly used anode material because of its high electrical conductivity, cycling stability, and reversible storage capacity for Li ions. However, graphite as an anode material has a limited theoretical capacity (372 mAhg^−1^) [10,11,12,13]. For large-scale applications, the next-generation anode must have a specific, stable capacity of at least 1000 mAhg^−1^ [14]. Silicon (Si) is the best element for the replacement of graphite anode materials because it has a high theoretical weight per unit capacity equal to 4200 mAhg^−1^ at very low potentials. The large difference in capacity between silicon and graphite occurs because silicon atoms can bind up to four lithium ions (chemically Li_4.4_Si/Li_22_Si_5_), while six carbon atoms can combine with each lithium ion [15,16,17,18].

However, the application of silicon as an electrode material has certain disadvantages. The most significant drawback is that as lithium alloys with silicon, silicon expands to approximately 400% of its original size, which then further expands to various sizes during de-lithiation. This phenomenon has previously been revealed using imaging techniques [19,20,21]. The expansion induces stress and deformation in the silicon particles, causing them to crack and break; this process of silicon decomposition is called grinding. Because of this effect, silicon is gradually peeled off the current collector and, thus, can no longer function as an electrode material. In addition, the space created by the expansion pushes the material surrounding the conductive material away from it, thus causing contact loss and a decrease in the electrical conductivity. In the absence of a strong electrical contact with the current collector, the silicon fragments will neither alloy with lithium nor contribute to the capacity of the battery. This behavior results in decreased stability and reversible capacity over several cycles.

In this study, it is demonstrated that silicon nanoparticles distribute evenly within the carbon matrix. Their presence in deeper regions within the internal structure could contribute to the prevention of these problems. Generally, the synthesis of silicon–carbon composites involves multiple steps: (i) synthesis of silicon nanoparticles, (ii) synthesis of carbon materials, and (iii) dispersion of nanoparticles within the carbon material. Various methods have been devised for the synthesis of silicon nanoparticles, including electrochemical etching [22,23], annealing of the borophosphosilicate glass [24,25], and thermal plasma treatment [26,27]. However, these methods require multiple steps or expensive and unique mechanisms. The additional chemicals introduced in this process result in high capital costs and add impurities in the synthesized particles.

In addition to the above, when the carbon synthesis process is added and nanoparticles are dispersed inside the carbon, the advantage associated with the reduction of the dimension of the synthesized product in mass production schemes is lost. Moreover, the use of a common dispersion method makes the achievement of an even distribution in the carbon matrix difficult.

Therefore, it is anticipated that the aforementioned drawbacks in multiprocessing and dispersion will be overcome if all of these processes are completed in one step, and the synthesis of silicon nanoparticles and carbon materials simultaneously occurs when a bottom-up approach is used. In this study, we synthesized a silicon–carbon matrix (Si–C) using a new method called solution plasma process (SPP). Silicon and carbon were simultaneously synthesized by dissolving the silicon solution precursor in an organic solvent to generate plasma. The synthesized silicon nanoparticles and the carbon matrix were synthesized in a similar manner to carbon black, and it was confirmed that the silicon particles were evenly distributed in the carbon matrix. In addition, the electrochemical performance evaluation shows that Si–C exhibits stable cycling performance with a high, reversible capacity.

## 2. Materials and Methods 

### 2.1. Synthesis of Si–C Composite

**Figure 1:**
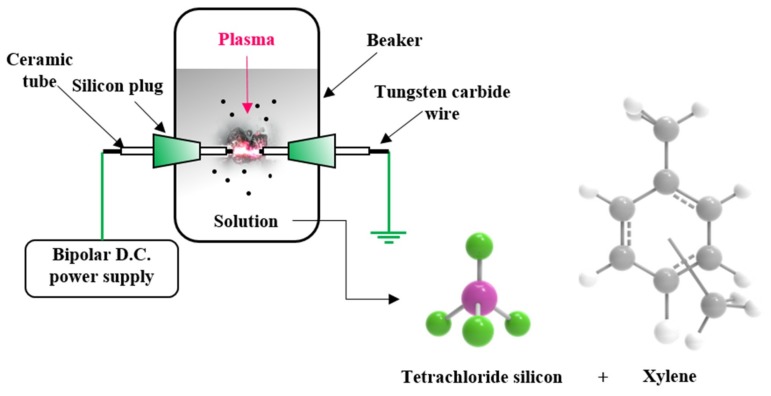
Schematic illustration of the solution plasma process (SPP).

A tungsten carbide wire with a diameter of 1 mm was used to generate plasma in solution. To concentrate the energy at the end of the wire, the wire was wrapped in a ceramic tube with an outer diameter of 2 mm and an inner diameter of 1 mm, and only 1 mm of the wire end was exposed. Silicon tetrachloride and xylene were used as silicon and carbon precursors, respectively, and a solution was prepared by mixing them in a 1:1 ratio. A pair of holes was machined in the middle of a beaker, a pair of tungsten carbide electrodes was placed in the holes, and the solution was added. A bipolar direct current (DC) pulsed power supply (Kurita, Japan) was then used to discharge the plasma in solution (Figure 1). The experiment was carried out at 25 °C under atmospheric pressure with a 2.0 kV voltage, 100 kHz frequency, and 1.0 µs pulse width applied to generate plasma. In order to increase the electrical conductivity of the synthesized carbon matrix and to remove remaining hydrogen, heat treatment was performed at 1000 °C in a tube furnace under a nitrogen atmosphere for 1 h (heating rate: 7 °C/min and furnace cooling). 

### 2.2. Material Characterization

The morphology of Si–C and the distribution of Si and C elements in the Si–C matrix were observed using transmission electron microscopy (TEM; TALOS F200X, Thermo Fisher Scientific, Loughborough, UK). In order to calculate the specific surface area of Si–C, N_2_ isothermal adsorption–desorption curves were measured (Autosorb iQ, Quantachrome Instruments, Boynton Beach, FL, USA) and analyzed using the Brunauer-Emmett-Teller (BET) method. Before the BET measurement, heat treatment was performed at 200 °C for approximately 2 h to completely remove moisture from the sample. The X-ray diffraction (XRD) profile was measured using an XRD D8 Discover (Bruker, Hamburg, Germany) with a Cu Kα (λ = 1.54 nm) target.

### 2.3. Electrode Manufacturing and Electrochemical Test

A 2032 coin-cell-type battery (Wellcos Corporation, Seoul, Korea,) was employed for the electrochemical performance testing of Si–C. The slurry for the anode electrode was prepared by dissolving Si–C, conductive carbon black (TIMCAL Graphite & Carbon Super P, Imerys, Paris, France), and the poly(acrylic acid) (average molecular weight 3,000,000) binder in distilled water at a weight ratio of 7:1:2. The slurry was uniformly mixed using an AR-100 conditioning mixer (THINKY Corporation) and uniformly coated on copper foil using a doctor blade. The copper foil was then dried under vacuum at 50 °C for 12 h to remove the solvent, then pressed with a roll press to increase the density and adhesion. The mass loading of the electrode material coated on the current collector was approximately 0.0032 g. A Li metal coin chip was used as the cathode. As a solvent for the electrolyte solution, a mixture of ethylene carbonate and dimethyl carbonate at 1:1 (v/v) was used; LiPF_6_ salt was dissolved to a concentration of 1 M and used as a solute. The electrolyte also contained 10 wt % fluoroethylene carbonate as an additive. Electrochemical tests were performed using a BCS-805 Biologic Battery Test System (Biologic, Seyssinet-Pariset, France).

## 3. Results and Discussion

**Figure 2:**
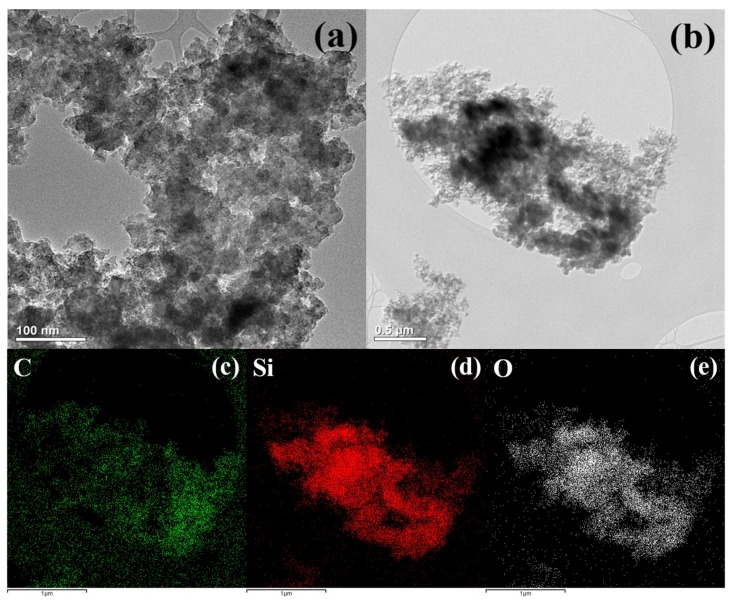
Transmission electron microscopy (TEM) images of (**a**) silicon nanoparticles and (**b**) silicon–carbon matrix (Si–C) composite via SPP. Energy dispersive spectroscopic (EDS) elemental mapping of (**c**) carbon, (**d**) silicon, and (**e**) oxygen corresponding to (**b**).

Figure 2a shows a transmission electron microscopy (TEM, TALOS F200X, Thermo Fisher Scientific, Loughborough, UK) image of Si nanoparticles synthesized when discharged with the use of a silicon tetrachloride solution. Si NPs have a regular chain-like spherical shape, and the aggregates are shaped like carbon black. In other words, small primary particles are connected to each other to form aggregate structures. The principle upon which Si NPs form agglomerates can be understood using diffusion-limited cluster aggregation (DLCA) models, such as carbon black. In other words, when many Si particles are made of plasma at the same time, they are interconnected in different directions as they diffuse randomly owing to Brownian motion. In this process, Si NPs become a three-dimensional network and a cohesive structure. On the other hand, the plasma generated in the SPP is very small compared with conventional vacuum plasma. Thus, even if Si NPs are generated, they quickly exit the plasma region due to the convective flow between the plasma, gas, and solution. Consequently, there is not enough time to grow larger particles, which results in smaller particles. The diameter of Si NPs ranged from 20 to 30 nm, and the aggregates were less than 1 μm. Chemical characterization of the carbon in the studied material has been performed in previous studies [28,29,30,31,32]. Figure 2b shows the TEM image and the energy dispersive spectrum (EDS) mapping results (Figure 2c,d) of the synthesized Si–C composite in a 1:1 solution of silicon tetrachloride and xylene. Both Si and carbon formed as carbon black, thus indicating that Si and carbon are composites in a well-dispersed form.

**Figure 3:**
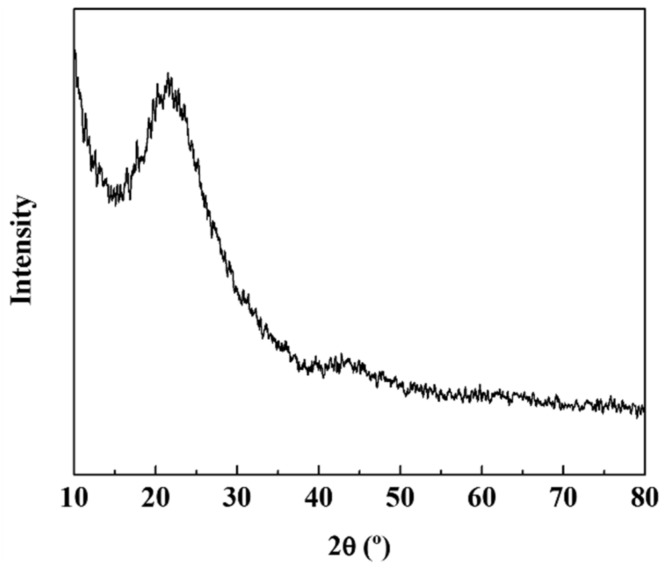
X-ray diffraction (XRD) patterns of Si–C.

Figure 3 shows the XRD pattern of the Si–C composite. The peak of the silicon carbide was not detected, which indicates that Si and C were present as separate materials. This outcome matches the EDS mapping results. However, it was presumed that silicon oxide was partially formed owing to its contact with oxygen during the synthetic process or electrode manufacturing. In the future, we expect that only pure silicon particles will be left if the electrode is fabricated with a complete shutdown of the atmosphere so that it can be oxidized. On the other hand, SiO_2_ forms non-active Li_2_O and lithium silicate during the reaction with lithium, acting as a buffer to mitigate the volume change of Si. Although the capacity of SiO_2_ is less than that of pure Si, it has been shown to improve the capacity retention characteristics. In this study, partially oxidized SiO_2_ functions as a buffer and shows capacity retention characteristics, which can be confirmed by electrochemical analysis.

**Figure 4:**
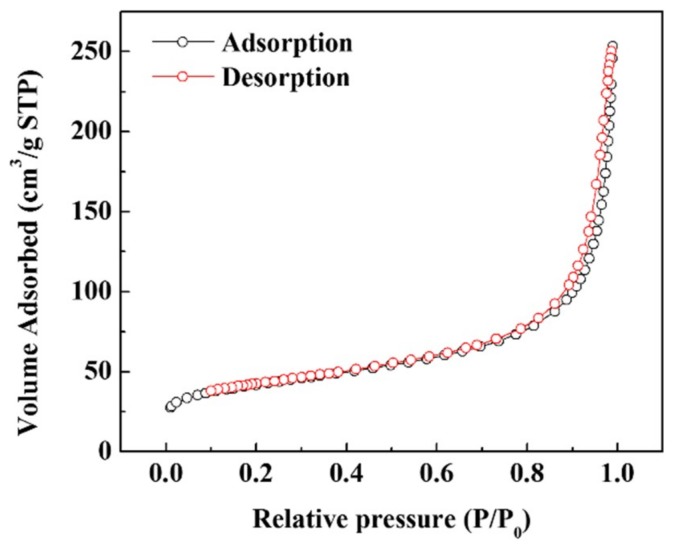
Nitrogen adsorption/desorption isotherms of Si–C.

The nitrogen adsorption/desorption isotherms of Si–C are shown in Figure 4, and the results are summarized in Table 1. A linear BET range of 0.05–0.35 was used, and a BET surface area of Si–C was calculated to be 8.2 m^2^ g^−1^. Given that both Si and carbon form a carbon black structure, the specific surface area is large. Because of this large surface area, the primary cycle efficiency may be lowered owing to the consumption of Li following the initial formation of the solid electrolyte interface (SEI) layer. However, these disadvantages can be overcome by the developed prelithiation process.

**Figure 5:**
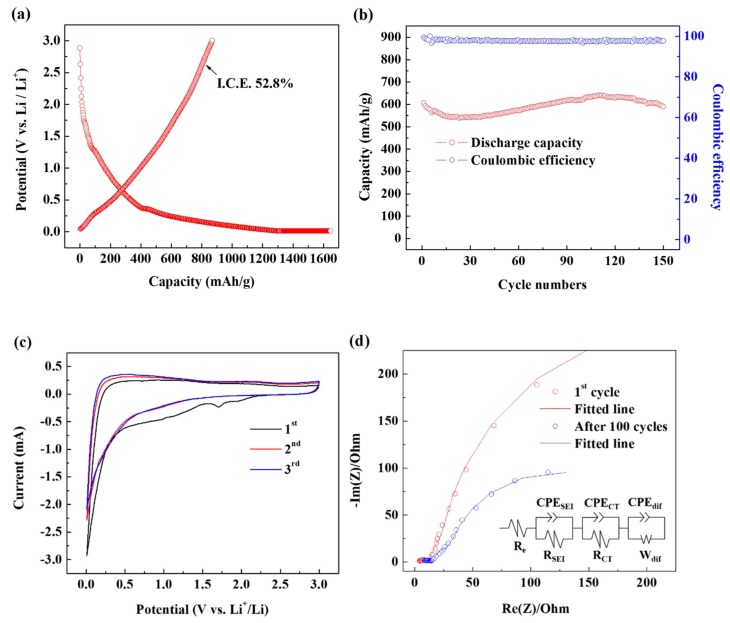

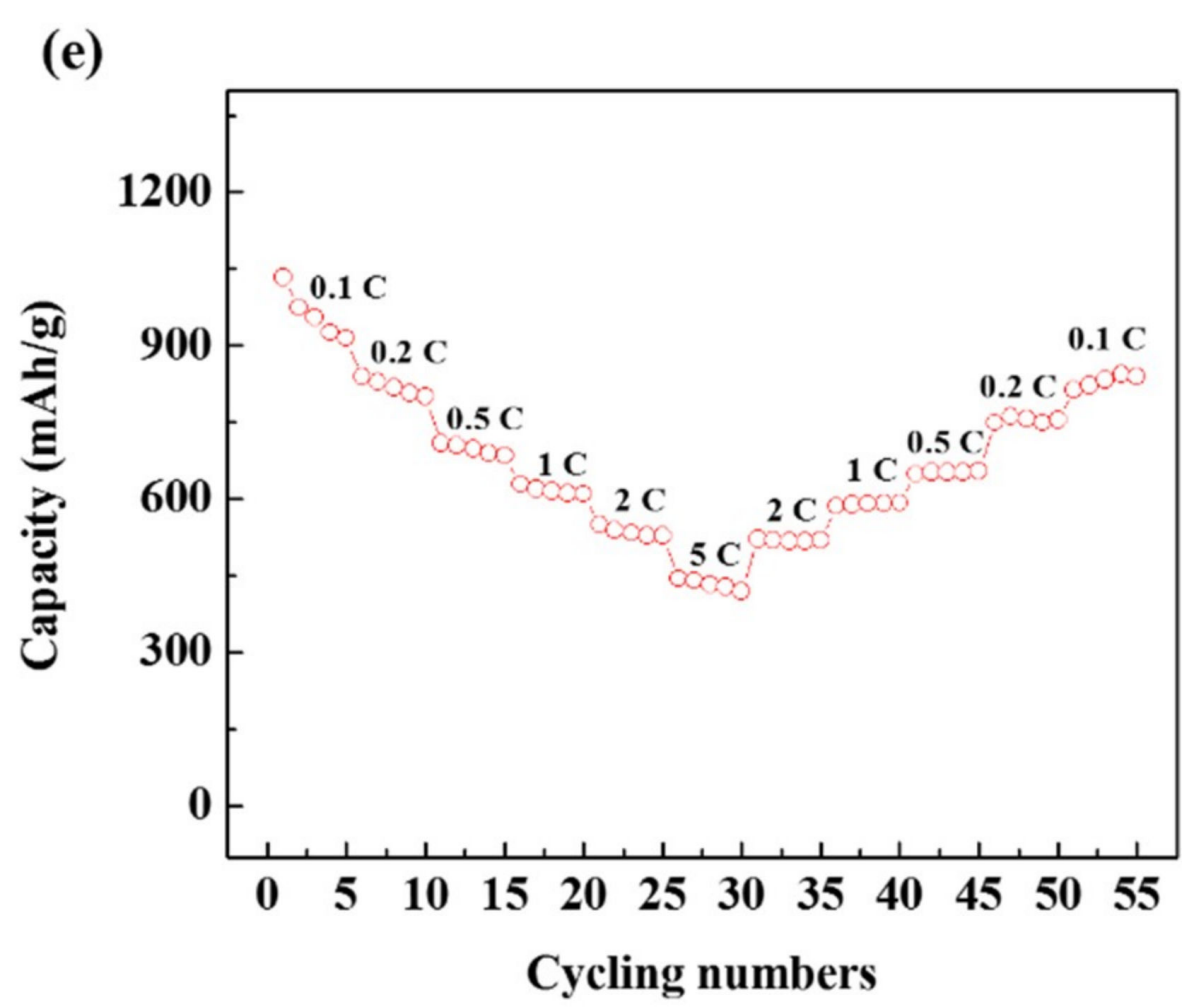
Electrochemical characteristics of the Si–C anode: (**a**) First cycle’s charge–discharge curves. (**b**) Cycling performance under 1 C (372 mA g^−1^). (**c**) Current–potential (CV) curves for the first three cycles. (**d**) Nyquist plots after several cycles. (**e**) Rate capability at various current densities from 0.1 C to 5 C.

Figure 5a shows the charge and discharge profiles of the first cycle of Si–C. Given that the specific surface area of the Si–C is large, a large SEI layer was formed, and the coulombic efficiency of the first period was 52%. However, it was completely recovered in subsequent cycling (Figure 5b) and yielded more than 99%, indicating that the formed SEI layer worked reliably.

Figure 5c shows the first three continuous cyclic voltammetry (CV) curves of Si–C measured in the voltage range of 0.01–3.0 V (vs Li/Li^+^) at a scan rate of 0.2 mV/s. The first peak at 1.1 V is the decomposition of fluoroethylene carbonate indicating that the additive functioned as intended. The second peak, which appears in the wide range of 0.25–1.1 V, is formed by the decomposition of ethylene carbonate and diethyl carbonate signifying the formation of an SEI layer. Finally, the sharp peaks observed below 0.25 V indicate that Li ions are alloyed with Si at the same time as they are inserted into the carbon matrix. In the second cycle, there is no peak from the decomposition of the electrolyte, indicating that the electrode surface is well covered by the SEI layer. In addition, the complete overlap of the second and third cycle curves shows that the insertion and desorption reactions of Li into Si–C are reversibly stable.

Impedance measurements were performed to study the charge transfer resistance and durability of the SEI films (Figure 5d). Here, *R*_e_ is the internal resistance of the electrolyte and electrode, *R*_SEI_ is the resistance of the SEI layer, and the constant phase element (CPE)_SEI_ is the capacitance of the SEI layer. CPE is the capacitance property that exists when the surface of the electrode is irregular [33,34,35,36]. In other words, Si–C has a carbon black structure, which has a large, specific surface area and complex shape, and exhibits CPE in electrochemical impedance spectroscopy (EIS) measurements. *R*_CT_ is the charge transfer resistance in the middle frequency region, *R*_dif_ is the Warburg resistance, and *C*_dif_ is the double-layer capacitance due to ion transport in the electrode material. Table 1 summarizes the parameters obtained from the EIS plot, showing that all resistance values slightly increased even after 100 cycles. This suggests the reliability of the SEI layer.

Figure 5e shows the C-rate capability of Si–C. The current density increased from 0.1 C to 5 C and then returned to 0.1 C, where 1 C is the current density corresponding to 372 mA/g. At 0.1 C, a very high reversible capacity of 900 mAhg^−1^ is observed, and at 1 C, a reversible capacity of about 600 mAhg^−1^ is shown. This corresponds to about twice the reversible capacity of a typical carbon material. At 2 C, Si–C shows a discharge capacity of about 550 mAhg^−1^, which is about 91% of that of Si–C at 1 C, demonstrating the excellent C-rate capability of Si–C. Reversible capacity is fully restored when the C-rate is back to 0.1 C after an increase, indicating that Si–C’s performance is stable at various current densities. 

## 4. Conclusions

Si–C composites were synthesized through a plasma process using Si and C as raw materials. TEM analyses showed that Si and C were similar to the carbon black structure in which small spherical primary particles aggregated to form a network. In addition, TEM, EDS and XRD analyses showed that Si and C were present as discrete materials rather than as silicon carbide compounds. Additionally, it was shown that Si was evenly and deeply distributed throughout the interior of the C matrix. This structure confirmed that the Si–C composite material exhibited a cycling performance of 150 cycles or more and a high discharge capacity as an anode material of the lithium-ion battery. As a result of BET analyses, the specific surface area of Si–C was considerably large, and it is thus expected that better performance can be achieved by optimizing the slurry formulation with a binder suitable for such a wide and specific surface area. The Si–C composites synthesized in this study show that this material can be synthesized in a one-step solution using a bottom-up approach. In the future, the combination of various Si and C precursors will lead to improved Si–C composites. We expect that the syntheses of composites will become possible.

## Figures and Tables

**Table 1:** EIS fitting parameters of 1st cycle and after 100 cycles.

	*R*_e_ (Ω)	*R*_SEI_ (Ω)	*R*_CT_ (Ω)
1st cycle	1.761	11.01	12.28
After 100 cycles	3.689	14.07	15.07
